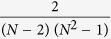# Corrigendum: A Stronger Multi-observable Uncertainty Relation

**DOI:** 10.1038/srep46949

**Published:** 2018-05-25

**Authors:** Qiu-Cheng Song, Jun-Li Li, Guang-Xiong Peng, Cong-Feng Qiao

Scientific Reports 7: Article number: 4476410.1038/srep44764; published online: 03 20 2017; updated: 05 25 2018

This Article contains errors in the following equations:

In Equation 7,
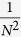


should read:
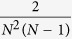


In Equation 9,
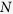


should read:
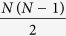


In Equation 10,
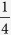


should read:
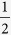


In Equation 16,
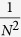


should read:
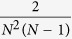


In Equation 17,
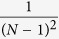


should read: